# *Burkholderia pseudomallei*: Its Detection in Soil and Seroprevalence in Bangladesh

**DOI:** 10.1371/journal.pntd.0004301

**Published:** 2016-01-15

**Authors:** Md. Shariful Alam Jilani, Jamshedul Alam Mohammad Robayet, Md. Mohiuddin, Md. Rokib Hasan, Chowdhury Rafiqul Ahsan, Jalaluddin Ashraful Haq

**Affiliations:** 1 Department of Microbiology, Ibrahim Medical College, Dhaka, Bangladesh; 2 Department of Microbiology, University of Dhaka, Dhaka, Bangladesh; 3 Department of Microbiology, Bangladesh Institute of Research and Rehabilitation in Diabetes, Endocrine and Metabolic Disorders, Dhaka, Bangladesh; University of Tennessee, UNITED STATES

## Abstract

**Background:**

Melioidosis, caused by *Burkholderia pseudomallei*, is an endemic disease in Bangladesh. No systematic study has yet been done to detect the environmental source of the organism and its true extent in Bangladesh. The present study attempted to isolate *B*. *pseudomallei* in soil samples and to determine its seroprevalence in several districts in Bangladesh.

**Methodology and Results:**

Soil samples were collected from rural areas of four districts of Bangladesh from where culture confirmed melioidosis cases were detected earlier. Multiple soil samples, collected from 5–7 sampling points of 3–5 sites of each district, were cultured in Ashdown selective media. Suspected colonies of *B*. *pseudomallei* were identified by biochemical and serological test, and by polymerase chain reaction (PCR) using 16s rRNA specific primers. Blood samples were collected from 940 healthy individuals of four districts to determine anti- *B*. *pseudomallei* IgG antibody levels by indirect enzyme linked immunosorbent assay (ELISA) using sonicated crude antigen. Out of 179 soil samples, *B*. *pseudomallei* was isolated from two samples of Gazipur district which is located 58 km north of capital Dhaka city. Both the isolates were phenotypically identical, arabinose negative and showed specific 550bp band in PCR. Out of 940 blood samples, anti- *B*. *pseudomallei* IgG antibody, higher than the cut-off value (>0.8), was detected in 21.5% individuals. Seropositivity rate was 22.6%-30.8% in three districts from where melioidosis cases were detected earlier, compared to 9.8% in a district where no melioidosis case was either detected or reported (p<0.01). Seropositivity increased with the advancement of age from 5.3% to 30.4% among individuals aged 1–10 years and > 50 years respectively. The seropositivity rates were 26.0% and 20.6% in male and female respectively, while it was 20–27% among different occupational groups. No significant association was observed with gender (χ2 = 3.441, p = 0.064) or any occupational group (χ^2^ = 3.835, p = 0.280).

**Conclusion:**

This is the first study demonstrating the presence of *B*. *pseudomallei* in the environmental (soil) samples of Bangladesh. It also suggested that a large proportion of people, residing in these districts, were exposed to the organism.

## Author Summary

Melioidosis, caused by *B*. *pseudomallei*, can be a fatal disease if not treated with appropriate antibiotics. The organism is mainly present in soil and water in endemic areas, and people become infected through skin inoculation, inhalation or ingestion. The disease has been sporadically detected in Bangladesh over last several decades. However, its actual prevalence in Bangladesh is largely unknown due to the lack of systematic study and awareness of the medical community about the disease and the organism. In order to address the issue, we have undertaken this study to assess the presence of the organism in the soil as well as its magnitude of exposure among the people of selected areas of the country. The study revealed the presence of *B*. *pseudomallei* in the soil and its exposure among the people of different areas. The information would increase the awareness of the medical community for prevention and correct diagnosis of the disease.

## Introduction

Melioidosis is an endemic disease of public health and clinical importance in tropical and subtropical regions of the world [1]. It is caused by a Gram negative saprophytic bacterium called *Burkholderia pseudomallei*. Infection occurs through skin and by inhalation when susceptible individuals are exposed to contaminated water and soil [2,3]. Melioidosis accounts for about 20% of all community-acquired septicemias in north-eastern Thailand and 2000 to 3000 new cases are diagnosed every year [4,5]. Multiple cases have also been reported from India and several countries of South-East Asia, the Middle East, Africa and South America [6].

In 1964, melioidosis was reported in a foreign sailor who was travelling through Bangladesh [7]. However, the first case of melioidosis in Bangladesh was diagnosed in a native Bangladeshi infant in 1988 [8]. Later on several cases of melioidosis were reported up to 2014 [9]. From 1991 to 1999, five cases were detected in United Kingdom (UK) among Bangladeshi people who immigrated to UK from the Sylhet region (a northeastern district) of Bangladesh [10–12]. In 2001, we reported the second culture confirmed suppurative melioidosis case in a 48 years old diabetic patient who came from Sherpur district of Bangladesh [13]. The district is located about 140 km north of capital Dhaka. Later on, at least 10 cases were detected among the diabetic patients at Bangladesh Institute of Research and Rehabilitation in Diabetes, Endocrine and Metabolic Disorders (BIRDEM) Hospital, Dhaka from 2009 to 2014 [9]. Analyses of the reported cases strongly indicate that the disease is potentially endemic in ten districts of Bangladesh particularly in northern and northeastern parts of the country.

Recently in 2013, melioidosis endemic countries of the world have been categorized into ‘definite’ and ‘probable’ country based on the presence of *B*. *pseudomallei* in humans and in the environment in the respective countries [6]. According to the above categorization, Bangladesh falls into ‘probable’ category of country as the presence of the organism in the environment has not yet been identified or reported even though several culture-confirmed melioidosis cases have been detected. Probability of the presence of *B*. *pseudomallei* in soil and water of Bangladesh is very high as the climatic condition of the country is favorable for its growth in the environment. Therefore, isolation and identification of *B*. *pseudomallei* from environmental samples (e.g. soil or water) is important to determine the source of the organism of melioidosis cases in the country.

The true extent of the disease in Bangladesh is not known, as this disease is not familiar to most of the physicians and microbiologists of the country. Seroepidemiological studies showed that 80% of children in north-eastern Thailand were positive for antibodies against *B*. *pseudomallei* by the age of 4 years [1]. In Malaysia, reported seroprevalence in healthy individuals was 17–22% among rice farmers and 26% in blood donors [14]. In north Australia 0.6 to 16% of children had evidence of infection by *B*. *pseudomallei* [15]. A hospital based serological survey in Bangladesh reported 28.9% seropositive rate for *B*. *pseudomallei* antibody among patients attending several tertiary care hospitals for unrelated ailments. The study, however, used a very low cut off titer (1:10) of indirect haemagglutination assay (IHA) for defining seropositive cases without considering the presence of cross reactive background antibody among the local population. The study did not investigate the possible source of the organism [16].

In view of the above, detection of *B*. *pseudomallei* in the soil samples and determination of anti-*pseudomallei* antibody in healthy population would help to establish the environmental source of the organism as well as the extent of its exposure in Bangladesh. So far, no systematic study has been done to find out the presence of organisms in environmental samples of Bangladesh. Therefore, the present study was designed for detection of *B*. *pseu*domallei by culture and molecular method from soil as well as to determine the extent of exposure by detecting antibodies to *B*. *pseudomallei* among the healthy population of four districts of Bangladesh.

## Materials and Methods

The present study was carried out to isolate and identify *B*. *pseudomallei* in soil samples from four selected geographical areas of Bangladesh. The study also determined the presence of anti- *B*. *pseudomallei* IgG antibody to find out the seroprevalence of the infection in four districts of the country. *B*. *pseudomallei* was isolated in selective media, identified by biochemical tests, and finally confirmed by specific antisera and PCR. Serum IgG antibody to *B*. *pseudomallei* was determined by ELISA. Details of the methods are described below.

### Ethics statement

The Ethical Review Committee (ERC) of the Diabetic Association of Bangladesh (BADAS) has approved the study. Ibrahim Medical College (IMC) is an institution under the BADAS and its ERC is the approval body for research protocols of IMC. Informed written consent was obtained from all adult participants (age 18 years and above) and from the parents/guardians of all children (age up to 17 years) prior to collection of blood samples and demographic data.

### Selection of sites and collection of soil samples

**Fig 1:**
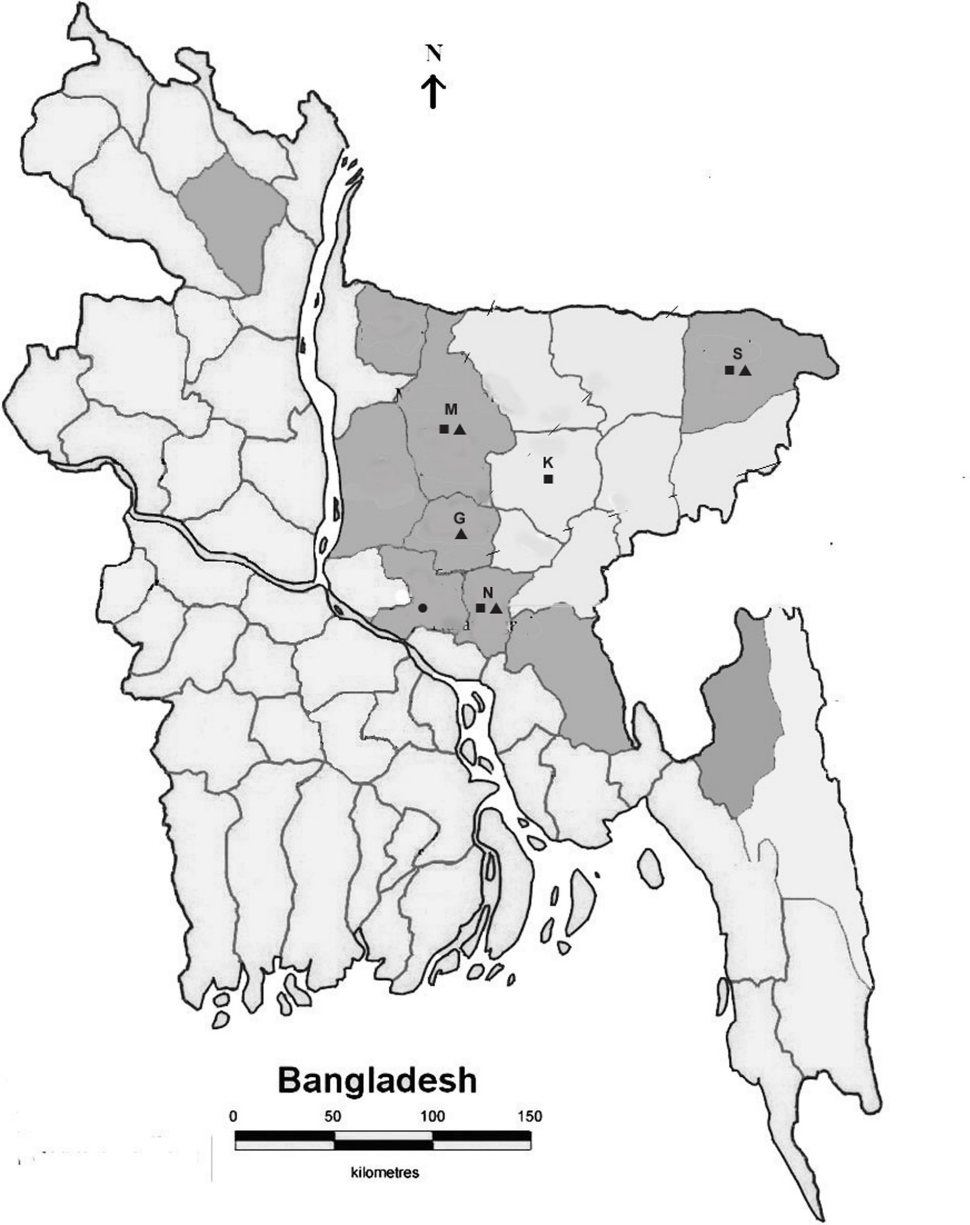
Map of Bangladesh showing collection sites (districts) of soil and blood samples. Shaded areas indicate districts with known melioidosis cases. M = Mymensingh, G = Gazipur, K = Kishoregange, S = Sylhet, N = Narayangange. The distance of Mymensingh, Gazipur, Kishoregange, Sylhet and Narayangange from capital Dhaka is 115, 40, 89, 198 and 14 km respectively. ■ = Site of soil collection; ▲ = Site of blood collection; ● = Capital Dhaka. Source of the map: [9].

Soil samples were collected from rural areas of four districts of Bangladesh with diagnosed melioidosis cases [9]. Three districts namely Mymensingh, Sylhet and Gazipur are situated in the north and northeast of capital Dhaka city while one district (Narayangange) is located south of Dhaka city. The locations of soil sampling district and their distance from capital Dhaka is shown in Fig 1. In each district, 3–5 sites were selected for collection of soil samples. Each sampling site was about 5 km apart from the next sampling sites. At each site 5-7sampling points were identified which were about 30 meters apart from each other. The preferred collection site was moist area within a rice field. Approximately 200 g soil was taken from each point from a depth of about 20–30 cm using a shell augur disinfected with 70% alcohol in between soil collection. Collected soil was placed into a sterile plastic bag and sealed with rubber band to prevent moisture loss and was transported to the laboratory as soon as possible. All the soil samples were collected and processed for culture from June to September 2011.

### Isolation and identification of *B*. *pseudomallei* from soil samples by culture

Soil samples were processed for culture as described by Brook et al [17]. Twenty grams of soil were mixed with 40 ml sterile distilled water and the suspension was shaken vigorously for one minute and allowed to settle for 5–10 minutes. The supernatant fluid was collected. For enrichment, 1 ml of supernatant fluid was inoculated into 9 ml of modified Ashdown’s selective enrichment broth (ASB) and incubated at 37^°^C for 48 hours [18]. After enrichment, 10 μl of broth was streaked onto modified Ashdown`s selective agar (ASA) medium. The plates were incubated for 48–72 hours to allow typical colonies to grow. Purple colored dry, wrinkled and oxidase positive colonies were then sub cultured on MacConkey`s agar medium and incubated at 42^°^C. The organisms which grew on MacConkey`s agar medium at 42^°^C were identified as *B*. *pseudomallei* by typical colony morphology, Gram staining (bipolar staining), motility, biochemical tests (including API 20NE), arabinose assimilation and resistance to colistin and aminoglycoside [19]. Monoclonal antibody based latex agglutination test (Melioidosis Research Center, Khon Kaen, Thailand) was performed for the final identification and confirmation of the suspected colonies of *B*. *pseudomallei*.

### Confirmation of *B*. *pseudomallei* by PCR

Serologically confirmed *B*. *pseudomallei* isolates from soil samples were further confirmed by PCR using specific primers constructed from 16s rRNA region of *B*. *pseudomallei* [16]. The primers were constructed from 16s rRNA region of *B*. *pseudomallei* to amplify a fragment of 550 bp in length. Primers were—PPM3 forward primer (5`AATCATTCTGGCTAATACCCG 3`) and PPM4 reverse primer (5`CGGTTCTCTTTCGAGCTCG 3`).

Total genomic DNA was prepared by RealLine DNA Extraction Sample Kit” (BIORON Diagnostics GmbH, Germany). Briefly, 500 μl preheated (at 56^°^C) lysis reagent with sorbent was added to 100 μl bacterial suspension. The tube was vortexed for 10 seconds followed by incubation in thermo shaker for 10 minutes at 1300 rpm. DNA/RNA solution was added to the tube and again vortexed and centrifuged at 13000 x g for 5 minutes. Supernatant was discarded and Wash Solution No 1 was added to the tube, vortexed and centrifuged at 13000 x g for 5 minutes. Supernatant was again removed and 300 μl Wash Solution No 2 was added to the tube, vortexed and centrifuged at 13000 x g for 5 minutes. Supernatant was discarded and the pellet was dried by opening the cap for 2–3 minutes at room temperature. 200 μl specimen diluents was added to the air dried tube and vortexed vigorously for 10 seconds followed by incubation at thermo shaker for 10 minutes at 56^°^C at 1300 rpm and then centrifuged at 13000 x g for 1 minute. Supernatant containing DNA was finally collected and stored at -20^°^C until used.

PCR amplification was carried out in a 25 μl final volume containing 2.0 μl DNA, 2.5 μl 1 x PCR buffer, 1.5 mM MgCl_2_, 25 μM of each dNTP, 10 pM of each primer, and 1.25 unit of Taq DNA polymerase enzyme. Samples were subjected to initial denaturation at 94^°^C for 2 minutes followed by denaturation at 94^°^C for 60 sec, primer annealing at 55^°^C for 60 sec and extension at 72^°^C for 90 sec. Final extension was for 10 minutes at 72^°^C. Amplification was performed in Master Cycler (Eppendorf) programmed for 35 cycles.

Amplified PCR product was analyzed by electrophoresis in 1.5% agarose gel containing ethidium bromide (0.5 μg/ml) in TBE buffer (0.04 M Tris acetate, 0.001 M EDTA, (pH 8.6) and photographed under UV illumination. The bands were compared to the band obtained with a positive *B*. *pseudomallei* DNA control. In all assays, DNA from known *B*. *pseudomallei* was included as positive control. A tube without DNA served as no template DNA control. All suspected soil and clinical *B*. *pseudomallei* isolates from the present and previous study [9] were sent to the Emerging Pathogens Institute (EPI), University of Florida, USA for species identification using type III secretion system (TTS1) assay [20]

### Study population and collection of blood samples

Relatives or attendants of patients attending the rural healthcare facilities of the four districts namely Mymensingh, Sylhet, Narayangange and Kishoregange were recruited for determining the anti- *B*. *pseudomallei* antibodies (Fig 1). Blood samples were collected from 940 healthy individuals with no history of fever, persistent cough, wasting or suppurative lesion. Age, sex and socio-economic conditions were recorded.

In order to determine the cut off optical density (OD) value of ELISA test, 51 healthy newborn babies of Dhaka city were enrolled in the study. About 1–2 ml of venous blood was collected from each individual with proper aseptic technique. Serum samples from 10 culture confirmed melioidosis cases admitted at BIRDEM Hospital were included in this study as positive controls.

### Determination of anti- *B*. *pseudomallei* IgG antibody by ELISA

Serum anti- *B*. *pseudomallei* IgG antibody was determined by an indirect ELISA as described by Voller et al [21]. To prepare sonicated antigen, 50 ml of Trypticase Soya Broth (TSB) was inoculated with pure colonies of *B*. *pseudomallei* USM strain and incubated overnight at 37°C. Organisms were harvested by centrifugation for 30 minutes at 4000 x g at 10°C. Pellets were suspended with 3 ml of 25 mM Tris-HCL (pH 7.4) and washed three times with Tris-HCL for 30 minutes at 4000 x g at 10°C. Deposited pellet, suspended in 5 ml of ice-cold Tris-HCL, was sonicated at 40W for 8 minutes in each pulse inside the assigned biosafety cabinet. Sonicated bacterial suspension was then centrifuged at 5000 x g at 10°C for 30 minutes. After centrifugation, the supernatant containing the bacterial proteins was collected and its protein concentration was determined. The 96 well EIA plate (Linbro, USA) was coated with sonicated antigen 10 μg/ml in 0.5 M carbonate/bicarbonate buffer (pH 9.6). To each well 100 μl volume of coating buffer was added and incubated overnight at 4°C. The plate was washed three times with PBS-0.05% Tween 20 (PBS-T, pH 7.4)) and blocked by incubating for 2 hrs with PBS-T containing 2% BSA at 37°C. The plate was then washed three times with PBS-T. A volume of 100 μl serum (1:1600 dilutions) sample was added into each well and incubated for 4 hours at 37°C. After washing with PBST three times, 100 μl of horseradish peroxidase conjugated anti-human IgG antibodies (1:4000) was added and incubated at 37°C for 2 hours. After washing three times with PBST, 50 μl of TMB substrate was added to each well and incubated at room temperature for 30 minutes in dark. Then 50 μl of 1 M sulfuric acid was added in each well. The colour developed was measured by EIA plate reader (Human ELISA Reader) at 450 nm. Optimum concentration of the antigen (10 μg/ml) and serum dilution (1:1600) was predetermined by checkerboard titrations.

A cut off OD values for anti- *B*. *pseudomallei* IgG antibody was determined to find out the exposure rate to *B*. *pseudomallei* in the study population. ELISA was performed with sera from 51 healthy newborn babies of Dhaka city who were presumed not to be exposed to *B*. *pseudomallei*. The mean OD + 3xSD of these sera were taken as cut-off OD value to determine the exposure rate. The mean OD±SD of the 51 healthy newborn babies were 0.2±0.2. Therefore, the calculated cut-off OD value was 0.8 (0.2+3x0.2). Any sample showing OD above this cut-off value of 0.8 was considered positive and referred to as exposed to *B*. *pseudomallei* infection. The mean OD value of ten culture positive cases (positive control) was 2.26±0.2.

**Fig 2:**
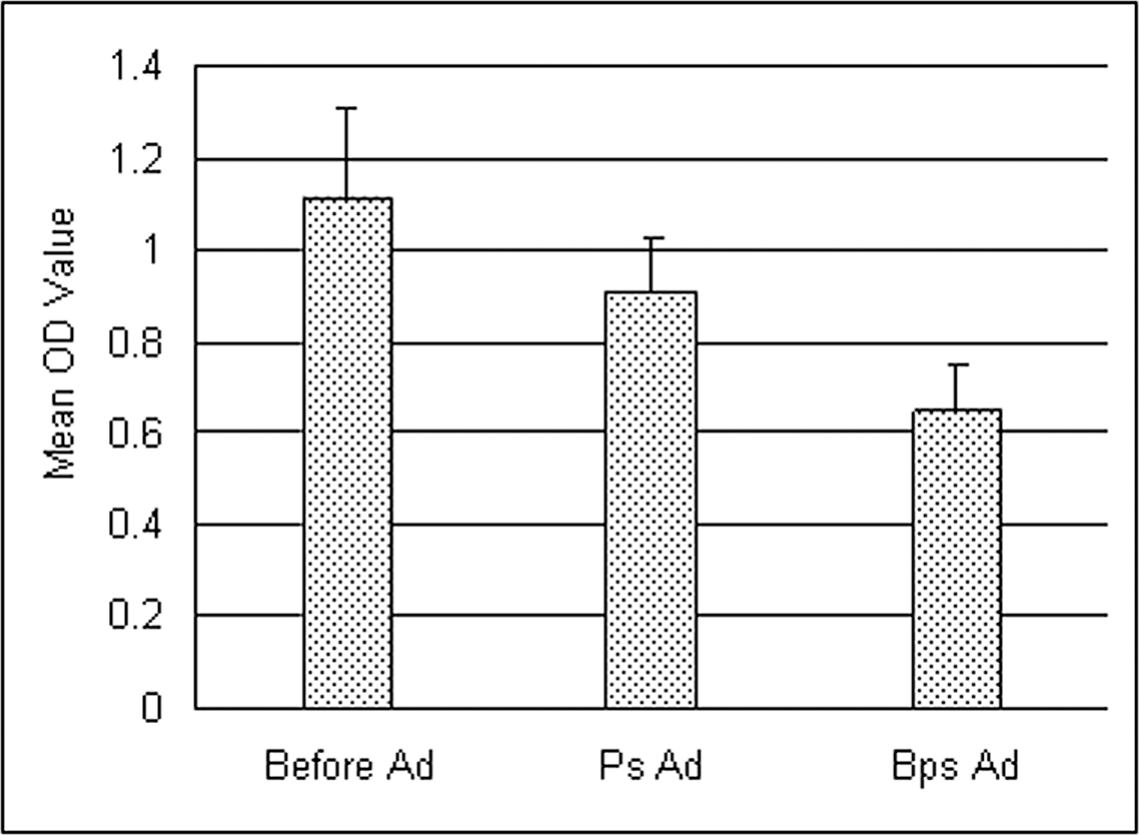
The effect of adsorption with whole cell *P*. *aeruginosa* and *B*. *pseudomallei* on the anti- *B*. *pseudomallei* IgG antibodies. Before Ad = Before adsorption; Ps Ad = Adsorption with *P*. *aeruginosa*; Bps Ad = Adsorption with *B*. *pseudomallei*. n = 24 seropositive sera in each group.

To determine the specificity of anti- *B*. *pseudomallei* IgG by ELISA, a sub-set of 24 known positive serum samples were adsorbed with whole cell killed *Pseudomonas aeruginosa* and *B*. *pseudomallei* USM strain (1x 10^8^ organisms/ml) by incubating overnight at 4^°^C. The adsorbed serum samples were centrifuged at 10,000 x g for 5 minutes to remove the bacteria. Anti- *B*. *pseudomallei* IgG antibody was then determined in adsorbed serum by ELISA as described above. Decline of antibody concentration in terms of OD values after adsorption with *B*. *pseudomallei* indicated presence of specific antibody to *B*. *pseudomallei* in serum samples while decline with *P*. *aeruginosa* indicated antibodies cross reacting to pseudomonas antigens. The adsorption assay showed that mean antibody level of the positive sera reduced significantly, after adsorption with *B*. *pseudomallei* compared to pre-adsorbed value from OD 1.1 to 0.6 (Fig 2). The mean OD value decreased below the cut off OD of 0.8 following absorption. But, the OD value decreased insignificantly from 1.1 to 0.9 following adsorption with *P*. *aeruginosa*

## Results

### *B*. *pseudomallei* in soil sample

**Fig 3:**
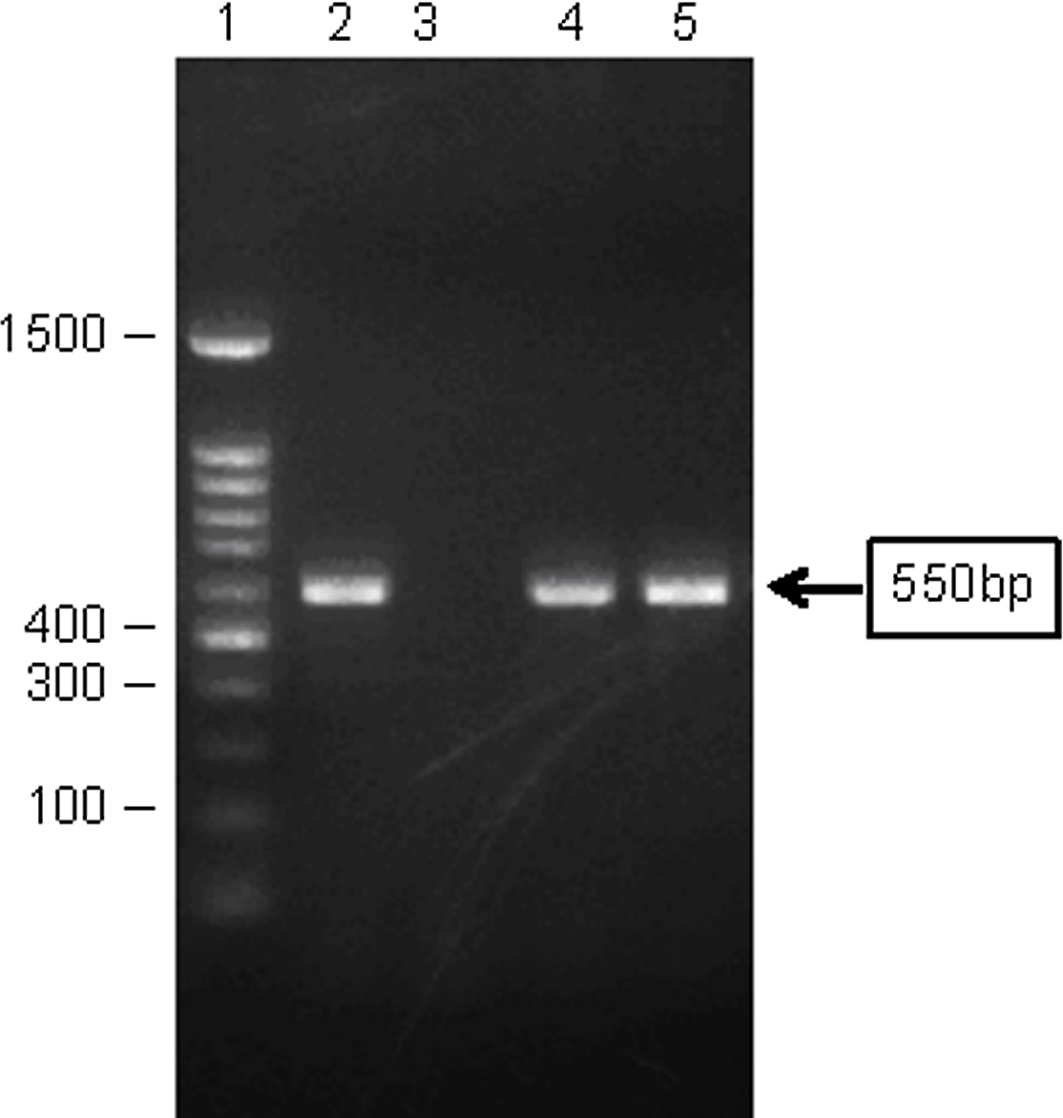
PCR analysis of *B*. *pseudomallei* isolates from Gazipur district. Lane 1- 100bp Marker, Lane 2- Positive control (USM strain of *B*. *pseudomallei*, Lane 3- Negative control (distilled water), Lane 4- K23 (Gazipur soil isolate) and Lane 5- K35 (Gazipur soil isolate).

Total 179 samples from four districts with diagnosed melioidosis cases were tested for the presence of *B*. *pseudomallei*. Out of 179 soil samples, 87 yielded growth of oxidase positive non-fermenting, aminoglycoside and colistin resistant suspected colonies on the Ashdown selective media after enrichment at 42°C. Out of these suspected isolates, only two isolates (K23 and K35) were identified as *B*. *pseudomallei* (Table 1). These two isolates were finally confirmed as *B*. *pseudomallei* by specific monoclonal anti-sera to *B*. *pseudomallei* and polymerase chain reaction (Fig 3 PCR gel). These two soil and all the clinical isolates (ten) were positive for TTS1 (S1 Table and S1 Fig). Both the isolates were arabinose negative suggesting that they were not *B*. *thailandensis*. The two soil samples that yielded growth of *B*. *pseudomallei* were collected from paddy field of Gazipur district. No other soil samples from any location yielded growth of *B*. *pseudomallei*. The remaining suspected isolates were identified as 12 different organisms (S2 Table).

**Table 1:** Results of culture of soil samples for the detection of *B*. *pseudomallei* from four different melioidosis endemic districts of Bangladesh.

Place (Districts)	No. of Sites	No. of Sampling points	Location of sampling points	No of Samples	No. of suspected isolates	No. positive for *Bps*
Gazipur	4	7	4 paddy field, 1 cattle shed, 1 poultry shed, 1 riverbank	41	15	2
Mymensingh	3	5	All paddy field	26	4	0
Narayangange	5	5	All paddy field	31	8	0
Sylhet	5	5	All paddy field	81	60	0
Total	19	22	-	179	87	2

Bps = B. pseudomallei

### Anti- *B*. *pseudomallei* IgG antibody in study population

Total 940 blood samples were collected from apparently healthy individuals residing in four districts of Bangladesh. Out of total 940 healthy subjects, anti- *B*. *pseudomallei* IgG antibody higher than the cut-off value (>0.8) were detected in 203 individuals (21.5%). Highest positive result was obtained from Mymensingh district (30.8%) while the rate was only 9.8% in Kishoregange district. The detail district wise rate of sero-positivity is shown in Table 2.

**Table 2:** Seroprevalence of anti- *B*. *pseudomallei* IgG antibodies among study population of four districts and mean OD values of positive and negative cases.

Place (Districts)	Total No. of Samples	Positive for anti- *B*. *pseudomallei* IgG		Mean (±SE) OD of	
		Number	% (95% CI)	Positive cases	Negative cases
Kishoregange	294	27	9.2 (5.8–12.5)	1.1 (0.04)	0.3 (0.01)
Mymensingh	221	68	30.8 (24.7–36.8)	1.2 (0.05)	0.3 (0.02)
Narayangange	214	58	22.6 (17.0–28.2)	1.4 (0.05)	0.4 (0.02)
Sylhet	211	50	27.5 (21.5–33.5)	1.1 (0.03)	0.4 (0.02)
Total	940	203	21.5 (18.9–24.9)	1.2 (0.03)	0.3 (0.01)

Cut off OD value for positive cases = 0.8

CI = Confidence interval

OD = Optical density

The age distribution of the seropositive cases showed that the maximum number (30.4%) of positive cases belonged to > 50 years age group while the lowest rate was among the 1–10 years age group (5.3%; Table 3). The seropositivity rate of anti- *B*. *pseudomallei* IgG antibody in male and female population and among different occupational groups ranged from 20% to 27% (Table 4). No significant association was observed.

**Table 3:** Age specific seropositivity rate of anti- *B*. *pseudomallei* IgG antibodies of study population.

Age group (years)	Total No of samples	Positive for anti- *B*. *pseudomallei* IgG		95% CI
		No	%	
1–10	57	3	5.3	0–11.1
11–20	89	20	22.7	13.9–31.3
21–30	153	22	14.4	8.9–19.8
31–40	174	25	14.4	9.2–19.6
41–50	214	56	26.2	20.3–32.0
>50	253	77	30.4	24.8–36.1
Total	940	203	21.5	18.8–24.1

Cut off OD value for positive cases = 0.8

CI = Confidence interval

**Table 4:** Distribution of occupation and sex of *B*. *pseudomallei* seropositive cases.

Characteristics	Total No of samples	Positive for anti- *B*. *pseudomallei* IgG		95% CI
		No	%	
Male	366	95	26.0	20.1–28.6
Female	523	108	20.7	16.3–23.0
House wife	473	97	20.5	16.8–24.2
Farmer	102	27	26.5	17.9–35.0
Service holder	137	33	24.1	16.9–31.2
Business	112	31	27.7	19.4–35.9
Others	115	15	13.0	6.9–19.1

No association with gender (χ2 = 3.441, p = 0.064) and with any occupational group (χ^2^ = 3.835, p = 0.280)

CI = Confidence interval

## Discussion

In Bangladesh, melioidosis has been infrequently detected for last the 25 years but no systematic epidemiologic information regarding its true magnitude and source is available. Isolation of the organism, *B*. *pseudomallei*, from clinical specimens indicates that the organism is present in our environment. However, its actual source has never been identified. The present study has been designed to isolate *B*. *pseudomallei* from the soil as well as to determine the seroprevalence among an apparently healthy population residing in four northern and northeastern districts of Bangladesh from where melioidosis cases were diagnosed previously.

In the first phase of the study, we aimed to find out the presence of *B*. *pseudomallei* in the soil samples from the melioidosis endemic districts of Bangladesh. This strategy of selecting four endemic districts to detect *B*. *pseudomallei* from environmental sources has been previously used throughout South-East Asia and northern Australia [22]. Out of 179 soil samples, only two soil samples (K23 and K35) from paddy fields of Gazipur district yielded growth of *B*. *pseudomallei*. Our failure to isolate *B*. *pseudomallei* from soil samples from other sites could be due to soil condition at the time of sample collection and limited number of samples. The presence of *B*. *pseudomallei* is influenced by low bacterial density, rain fall, load of organic materials and oxygen contents of the soil [23]. It is to be noted that the collection and culture of soil in our study was carried out from June to September 2011 which encompasses summer and rainy seasons. Moreover, if we could use the method of soil culture as outlined in international consensus guidelines of 2013 [6] then the rate of isolation of the organism might be higher than the observed result.

The isolation of *B*. *pseudomallei* from the soil samples in one district of Bangladesh indicates for the first time that the organism might be present in the local environment and is the source of human infections. However, we could not ascertain whether our soil and previous clinical isolates were similar and whether the organisms from the soil reservoir were the source of clinical infections. If we could perform multi locus sequence typing (MLST) or sequencing of the genome of the isolates then we could confirm that the soil organisms might be the source of the clinical infections in Bangladesh. But, the presence of the organism in the soil strongly indicates that it could be a potential reservoir. So far 18 countries of the world had been designated as definitive country for melioidosis based on the presence of culture confirmed *B*. *pseudomallei* in clinical case as well as in the environmental samples namely soil, water, etc of the locality [6,24]. Our finding of *B*. *pseudomallei* from the soil of one of the districts finally confirms that Bangladesh too is a definite endemic country for melioidosis.

In the second phase of the study, blood samples were collected from healthy population of four districts to determine the seroprevalence of *B*. *pseudomallei* infection. We have used ELISA assay using crude sonicated antigen to determine the presence of anti- *B*. *pseudomallei* IgG antibody. The rate of serological positive cases for anti- *B*. *pseudomallei* IgG antibody ranged from 22%-31% among study population of three districts where melioidosis cases were detected earlier. The seropositive rate was highest in Mymensingh (30.8%) district while it was lowest (9.2%) in Kishoregange district where no case of melioidosis has yet been diagnosed or reported. The serological findings suggest that there is a wide exposure of people to *B*. *pseudomallei* present in the soil or other environmental sources. Seroprevalence rates may vary widely according to the region within the endemic country [25]. Our overall seroprevalence rate was almost similar to rates reported from other countries of the region [26]. In 2012, a hospital based serological study in Bangladesh has reported sero-positive rate of 28.9% for *B*. *pseudomallei* antibody among various patients attending selective tertiary care hospitals of the Bangladesh [16]. The study used a very low cut off titer (1:10) of indirect haemagglutination assay (IHA) for defining seropositive cases. The use of the IHA in sero-epidemiological study is problematic in endemic areas, particularly where rates of background seropositivity may be high [26], presumably due to subclinical exposure to organisms related to *B*. *pseudomallei* [27,28].

It is important to note from our adsorption study that some degree of non-specific cross reactive antibody was present in the serum which reacted with crude whole cell antigen used in the ELISA as was seen by reduction of OD value following adsorption with *P*. *aeruginosa* antigen. This non-specific reactivity might contribute to higher rate of seropositivity. Therefore, we assume that the seropositivity rate could be a little lower if more defined and specific antigen from *B*. *pseudomallei* was used. Such specific antigen would eliminate the cross reaction with background antibody to related species of *B*. *pseudomallei* like *B*. *thailandensis* or *B*. *cepacia*.

In our study, seropositivity rate for *B*. *pseudomallei* antibody significantly increased with advancement of age. Seropositivity was highest (30.4%) among individuals more than 50 years of age. Lowest rate (5.3%) was observed in 1–10 years age group. The result suggests that the chance of exposure to *B*. *pseudomallei* increases with age. Most of the people residing in the rural settings are involved in agricultural work and frequently come in contact with soil, mud and contaminated water, as they grow older.

The rate of seroprevalence among male and female population varies in different studies [29,30]. However, we found no significant association of seropositivity with gender or any particular occupation. Probably our adult inhabitants in rural areas are equally exposed to the environment.

The present study has for the first time identified the presence of *B*. *pseudomallei* in the soil samples of Bangladesh. The study has also demonstrated that a large proportion of people residing in four districts are exposed to the organism and have a potential for developing overt diseases during their lifetime.

## Supporting Information

S1 Table Results of TTS1 assay by real time PCR. (DOCX)

S2 Table Name of other organisms isolated from the soil samples. (DOCX)

S1 Fig Graph showing TaqMan TTS1 real time PCR assay. (PDF)
