# Supplementary material for: Burkholderia pseudomallei: Its Detection in Soil and Seroprevalence in Bangladesh
Source: PLoS Negl Trop Dis. 2016 Jan 15;10(1):e0004301. doi: 10.1371/journal.pntd.0004301 (PMC4714902; doi:10.1371/journal.pntd.0004301)
Supplement: S1 Table — (DOCX) [file pntd.0004301.s001.docx]

S1Table: Results of TTS1 assay (real time PCR) for the confirmation

of *B. pseudomallei* isolated from clinical and soil samples

| Sample No | TTS1 TaqMan Assay |
| --- | --- |
| CS 14 | + |
| CS-27 | + |
| CS-35 | + |
| CS 55 | + |
| CS-88 | + |
| CS-90 | + |
| CS 1032 | + |
| CS-2327 | + |
| CS 4382 | + |
| CS-6318 | + |
| K23 (Soil) | + |
| K35 (Soil) | + |

Note: TTS1= Type III secretion system; CS= Clinical sample;

K= Kapasia; += Positive
