# Supplementary material for: Burkholderia pseudomallei: Its Detection in Soil and Seroprevalence in Bangladesh
Source: PLoS Negl Trop Dis. 2016 Jan 15;10(1):e0004301. doi: 10.1371/journal.pntd.0004301 (PMC4714902; doi:10.1371/journal.pntd.0004301)
Supplement: S2 Table — (DOCX) [file pntd.0004301.s002.docx]

S2 Table: Name of other organisms isolated from the soil samples

| Sl. No | Organisms |
| --- | --- |
| 1.  2.  3.  4.  5.  6.  7.  8.  9.  10.  11.  12. | *Burkholderia cepacia*  *Chrysobacterium meningosepticum*  *Comamonas testosteroni*  *Pseudomonas alcaligenes*  *Photobacterium damselae*  *Chromobacterium violaceum*  *Pseudomonas putida*  *Pseudomonas fluorescens*  *Achromobacter xylosoxidans*  *Aeromonas salmonicida*  *Chrysobacterium indologenes*  *Pseudomonas aeruginosa* |
