# Supplementary material for: Burkholderia pseudomallei: Its Detection in Soil and Seroprevalence in Bangladesh
Source: PLoS Negl Trop Dis. 2016 Jan 15;10(1):e0004301. doi: 10.1371/journal.pntd.0004301 (PMC4714902; doi:10.1371/journal.pntd.0004301)

S1 Fig: Graph showing TaqMan TTS1 real time PCR assay for confirmation of *B. pseudomallei* isolated from ten clinical and two soil samples

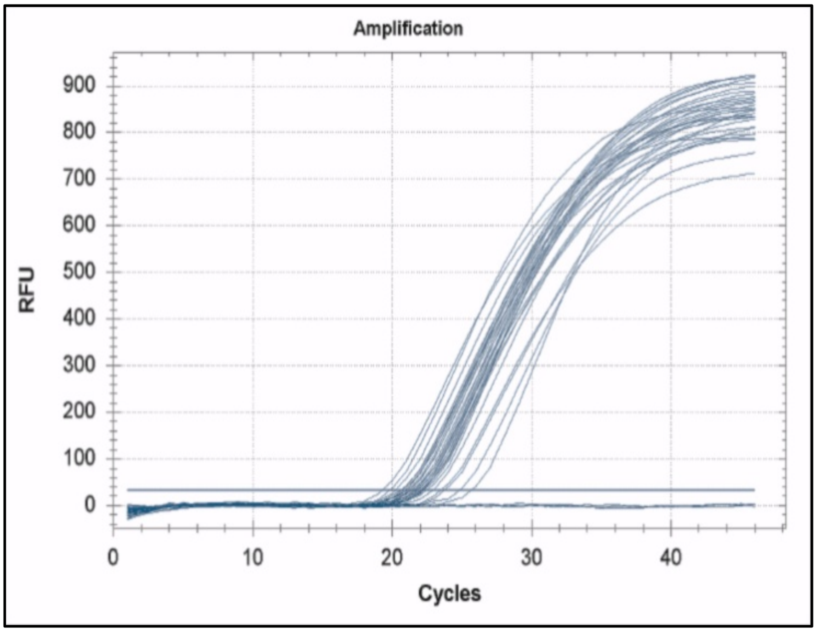

Supplement: S1 Fig — (PDF) [file pntd.0004301.s003.pdf]
